# Multimodality Imaging Methods for Assessing Retinoblastoma Orthotopic Xenograft Growth and Development

**DOI:** 10.1371/journal.pone.0099036

**Published:** 2014-06-05

**Authors:** Timothy W. Corson, Brian C. Samuels, Andrea A. Wenzel, Anna J. Geary, Amanda A. Riley, Brian P. McCarthy, Helmut Hanenberg, Barbara J. Bailey, Pamela I. Rogers, Karen E. Pollok, Gangaraju Rajashekhar, Paul R. Territo

**Affiliations:** 1 Eugene and Marilyn Glick Eye Institute, Department of Ophthalmology, Indiana University School of Medicine, Indianapolis, Indiana, United States of America; 2 Department of Biochemistry and Molecular Biology, Indiana University School of Medicine, Indianapolis, Indiana, United States of America; 3 Department of Pharmacology and Toxicology, Indiana University School of Medicine, Indianapolis, Indiana, United States of America; 4 Indiana University Melvin and Bren Simon Cancer Center, Indianapolis, Indiana, United States of America; 5 Stark Neurosciences Research Institute, Indiana University School of Medicine, Indianapolis, Indiana, United States of America; 6 Department of Cellular and Integrative Physiology, Indiana University School of Medicine, Indianapolis, Indiana, United States of America; 7 Eastern University, St. Davids, Pennsylvania, United States of America; 8 Department of Radiology and Imaging Sciences, Indiana University School of Medicine, Indianapolis, Indiana, United States of America; 9 Department of Medical and Molecular Genetics, Indiana University School of Medicine, Indianapolis, Indiana, United States of America; 10 Herman B Wells Center for Pediatric Research, Department of Pediatrics, Section of Pediatric Hematology/Oncology, Riley Hospital for Children at Indiana University Health, Indianapolis, Indiana, United States of America; 11 Indiana Center for Vascular Biology and Medicine, Indiana University School of Medicine, Indianapolis, Indiana, United States of America; Bascom Palmer Eye Institute, University of Miami School of Medicine, United States of America

## Abstract

Genomic studies of the pediatric ocular tumor retinoblastoma are paving the way for development of targeted therapies. Robust model systems such as orthotopic xenografts are necessary for testing such therapeutics. One system involves bioluminescence imaging of luciferase-expressing human retinoblastoma cells injected into the vitreous of newborn rat eyes. Although used for several drug studies, the spatial and temporal development of tumors in this model has not been documented. Here, we present a new model to allow analysis of average luciferin flux (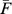) through the tumor, a more biologically relevant parameter than peak bioluminescence as traditionally measured. Moreover, we monitored the spatial development of xenografts in the living eye. We engineered Y79 retinoblastoma cells to express a lentivirally-delivered enhanced green fluorescent protein-luciferase fusion protein. In intravitreal xenografts, we assayed bioluminescence and computed 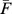, as well as documented tumor growth by intraocular optical coherence tomography (OCT), brightfield, and fluorescence imaging. In vivo bioluminescence, ex vivo tumor size, and ex vivo fluorescent signal were all highly correlated in orthotopic xenografts. By OCT, xenografts were dense and highly vascularized, with well-defined edges. Small tumors preferentially sat atop the optic nerve head; this morphology was confirmed on histological examination. In vivo, 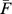 in xenografts showed a plateau effect as tumors became bounded by the dimensions of the eye. The combination of 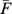 modeling and in vivo intraocular imaging allows both quantitative and high-resolution, non-invasive spatial analysis of this retinoblastoma model. This technique will be applied to other cell lines and experimental therapeutic trials in the future.

## Introduction

The pediatric ocular tumor retinoblastoma is the prototypic genetic cancer [1]. It is initiated in most cases by mutation of both alleles of the *RB1* gene, the first tumor suppressor gene to be cloned, although some retinoblastomas initiate without *RB1* mutation [2]. In recent years, genetic characterization of retinoblastomas beyond loss of *RB1* has provided multiple potential targets for therapeutic intervention (reviewed in [3]), including the oncogenes *KIF14* [4], *MYCN* [2], *E2F3* [5], *DEK* [5], *MDM4* [6] and *SYK* [7], the tumor suppressor cadherin-11 [8], and the oncomiR cluster 17∼92 [9]. However, targeted therapeutics for retinoblastoma have yet to transition into the clinic. Currently, the standard of care for this cancer involves laser therapy or cryotherapy for small tumors, often with systemic cytotoxic chemotherapy. Treatment of large tumors often requires enucleation of the eye or the use of external beam radiation; however, patients subjected to radiation therapy incur a lifetime risk of treatment toxicity [1]. As molecular targeted therapies become a possibility for retinoblastoma, effective animal models are needed for testing these therapies in vivo [10].

Although genetically modified mice are popular models for retinoblastoma, the complex derivation of such models and lack of some shared characteristics with the human tumor [11] have led to considerable interest in xenograft models of this cancer. In recent years, bioluminescence imaging (BLI) has been combined with orthotopic retinoblastoma xenografts to document tumor growth in vivo [12], [13]. One model involves intravitreal injection of luciferase-expressing Y79 retinoblastoma cells into the eyes of newborn (postnatal day 0, P0), wild type rats [12]. This neonate model offers two key advantages: 1) a developmentally appropriate host environment for these pediatric tumor cells, and 2) a naturally immunonaïve setting, circumventing the need for immunocompromised animals. To date, this model has been used for testing novel treatments such as topotecan/carboplatin and topotecan/vincristine combination therapies [12], [14], viral expression of interferon [15], a novel isoquinoline EDL-155 [16], and a histone deacetylase inhibitor MS-275 [17].

In retinoblastoma and numerous other cancer models [18], BLI has been used for longitudinal assessment of factors including primary tumor development, metastasis, residual disease, and recurrence [19]–[21]. Strengths of BLI for non-invasively following tumor progression include a tightly coupled biochemistry producing chemiluminescent emission [22], [23], stereospecific substrates [24], and reactions that are governed by Michaelis-Menten kinetics [22], [25]. Nonetheless, emission from implanted tumor cells is impacted by a multitude of factors including tissue pH [26]–[28], tissue oxygenation [29], tumor blood flow [30], substrate concentrations [31], and substrate extraction from the blood. Moreover, image acquisition and optical physics limitations in dense tissues at 530 nm (i.e., penetration, scatter, attenuation, etc.) have limited the advancement of high resolution and tomographic BLI. Interestingly, the eye as a model system sidesteps many of these biophysical limitations, and in fact serves as a collimator for BLI photons due to the transparent nature of the cornea and lens. Thus this system is an apt one for further development of BLI algorithms.

Despite the recent successes and demonstrated value of BLI in the context of imaging neonatal rat retinoblastoma xenografts, intraocular tumor growth in this system has not been documented visually, and peak luminescence has been used as a surrogate for tumor vitality in all studies to date. In the current study, we modified this orthotopic xenograft model to allow intraocular imaging of tumor growth and mathematical modeling of the luciferin flux, 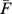, to yield more physiologically relevant measurements of viable tumor function over time.

## Materials and Methods

### Cell Culture

Y79 cells [32] were cultured in suspension in a humidified incubator at 5% CO_2_, 37°C. Growth medium was Iscove’s Modified Dulbecco’s Medium plus 10% “Gold” fetal bovine serum (PAA, Linz, Austria), 100 U/mL penicillin, 100 µg/mL streptomycin, 10 µg/mL insulin, and 55 µM β-mercaptoethanol. The identity of the Y79 cells (a kind gift of Brenda Gallie) was confirmed by short tandem repeat profiling and comparison to reference data (www.atcc.org).

The lentiviral vector, pCL6LucEGwo, encodes a fusion of the human codon usage-optimized luciferase (InvivoGen, San Diego, CA, USA) and enhanced green fluorescent protein (EGFP; Clontech, Mountain View, CA, USA) driven by a modified spleen focus-forming virus (SFFV) retrovirus U3 promoter [33]. Details on the vector and cloning will be available elsewhere (Wiek, Hanenberg, Pollok, submitted). Replication-incompetent infectious lentiviral particles in the vesicular stomatitis virus glycoprotein pseudotype (kindly obtained from Dirk Lindemann, Dresden, Germany) were generated using 293T cells as previously described [33] and high viral titers were obtained (∼10^9^ transduction units per mL). Overnight transduction of the cells with the supernatant resulted in significant toxicity. Therefore, a modified transduction procedure was used. Y79 cells were transduced for 4 hours with previously frozen supernatant at a multiplicity-of-infection of 50 in the presence of 8 µg/ml polybrene with no significant effects on cell viability. The transduction efficiency was determined by measuring EGFP expression by flow cytometry (BD LSR Cell Analyzer, BD Biosciences, San Jose, CA, USA). Approximately 99% of the Y79 cells were EGFP+.

### Cell-based Assays

For proliferation assays, Y79-EGFP-luc cells (2,500 cells) were seeded in 96-well black plates and alamarBlue added after the indicated times. Four hours after alamarBlue addition, fluorescence (λ_ex_ = 560 nm; λ_em_ = 590 nm) was measured on a Synergy H1 plate reader (BioTek, Winooski, VT, USA) and data analysis performed with GraphPad Prism and Microsoft Excel. An F-test was used to compare growth curves, with p<0.05 considered statistically significant.

For correlations between fluorescence, luminescence, and cell number, cells were manually counted by hemocytometer and the indicated cell numbers plated in 96-well black plates. EGFP fluorescence (λ_ex_ = 488 nm; λ_em_ = 528 nm) was measured on the plate reader, then a BrightGlo luciferase assay kit (Promega, Madison, WI) was used to measure luminescence on the plate reader according to the manufacturer’s instructions. Pearson’s correlation coefficients were calculated with Microsoft Excel.

To calculate ng luciferase per mg of cells, a population of Y79-EGFP-luc cells was counted by hemocytometer, yielding 3.06×10^7^ cells with 94% viability. These cells were pelleted into a tared microfuge tube and found to weigh 1.79 mg per million cells. The amount of luciferase per million cells was determined in quadruplicate using a Max Discovery Luciferase ELISA Kit (BIOO Scientific, Austin, TX, USA), yielding a value of 39.8 ng luciferase per million cells. The final conversion to ng luciferase per mg tumor was calculated using the aforementioned tumor cell weight, yielding 22.2 ng luciferase per mg tumor cells.

### Xenograft Generation

All experiments were approved by the Indiana University School of Medicine Institutional Animal Care and Use Committee (Protocol 10003) and adhered to all standards set forth in the ARVO Statement for the Use of Animals in Ophthalmic and Vision Research, including ensuring all efforts to minimize suffering. Animals were anesthetized with isoflurane and euthanized by isoflurane overdose followed by decapitation. A pregnant Sprague-Dawley dam (Harlan, Indianapolis, IN, USA) was housed individually on a 12-hour light-dark cycle (lights on at 0700) with access to food and water *ad libitum*. Within 24 hours of birth, newborn rat pups (P0) were anesthetized with isoflurane using an induction box attached to the anesthesia machine (3.0% isoflurane at an O_2_ flow of 1.5 L/min; Vetamac, Rossville, IN, USA). After induction, pups were tattooed on their paws for identification and then transferred to a heated surgical table to maintain body temperature at 37°C. Anesthesia was maintained (2.5% isoflurane at an O_2_ flow rate of 1.5 L/min) using a modified nosecone for rat pups.

**Figure 1:**
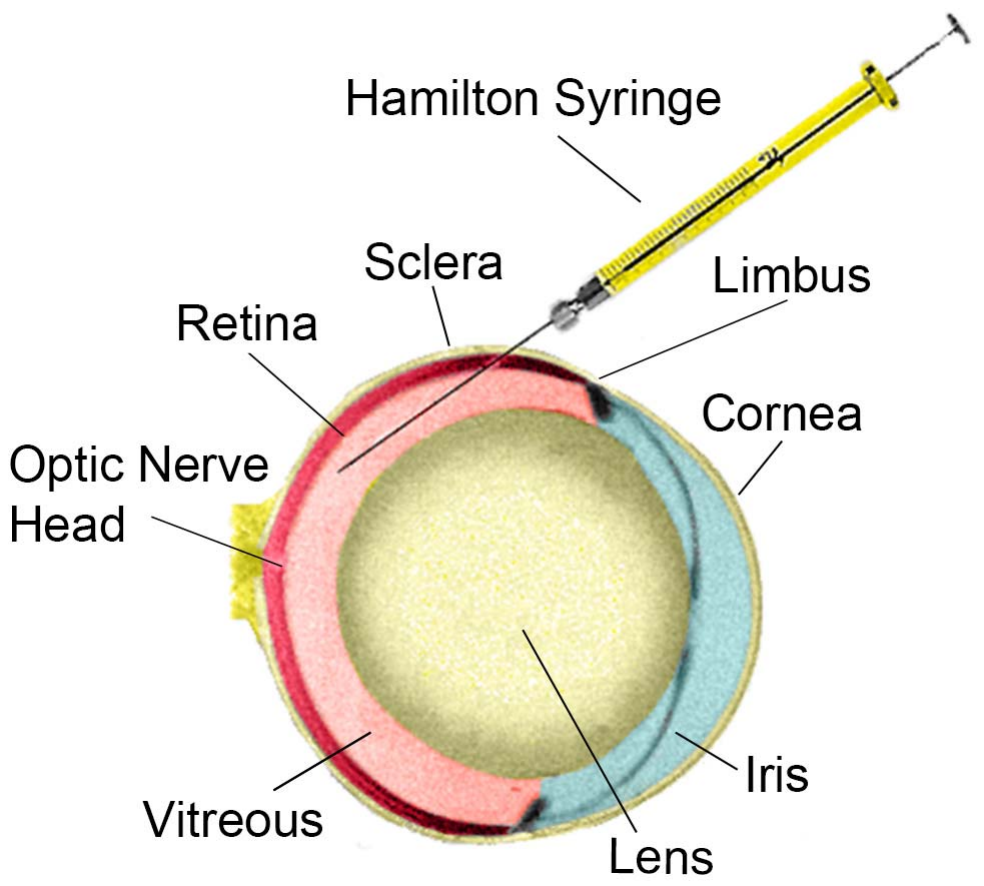
Illustration of the neonatal rat left eye, viewed from above, showing approximate injection angle and location at the lateral equator.

Under an operating microscope, Vannas scissors were used to open the eyelid fissure and an additional 2 mm lateral canthotomy was created for better visualization of the globe. To dilate the pupil, one drop of tropicamide 1% was placed on the operative eye. Colibri forceps were used to rotate the eye nasally and a 33G needle attached to a 5 µL Hamilton syringe was inserted bevel-up into the intravitreal space at the equator (Fig. 1). Each animal received a single injection of 10^3^ (n = 9) or 10^4^ (n = 6) Y79-EGFP-luc cells or a control injection of sterile phosphate buffered saline (PBS) vehicle (n = 3) into the right eye. Injection of cells or vehicle (1 µL total volume for all injections) was initiated by an assistant only when the tip of the needle was visualized through the dilated pupil by the surgeon. The cell suspension or PBS was injected over a 4–5 second period, and the needle remained in place for 1 minute following injection to minimize reflux when the needle was removed. Eyes were then thoroughly rinsed with sterile PBS, and pups were transferred to a homeothermic blanket until they awoke from anesthesia. Once awake, pups were returned to their mother in the home cage.

### Bioluminescence Imaging

Under isoflurane anesthesia performed as described above, animals (n = 6, 10^4^ cells injected per eye) were injected subcutaneously (s.c.) with VivoGlo d-luciferin potassium salt (Promega), from a 20 mg/mL stock in PBS. Dosage was 150 mg luciferin per kg animal weight; animals were weighed immediately prior to each imaging session. Under maintenance anesthesia, animals were imaged on the indicated days after injection (P3, P7, P10, P14) on a NightOwl LB981 (Berthold, Oak Ridge, TN, USA) system. Animals were imaged on their left sides, 3 at a time until too large for the chamber, then 2 at a time using full-frame camera height. Images were gathered over 6 intervals of 5 minutes each, for a total of 30 minutes’ imaging. In all cases, image import, visualization, segmentation and quantification were performed using custom, in-house developed bioluminescence image analysis software.

### Intraocular Imaging

Animals were allowed 2 weeks for natural eye opening before the intraocular imaging protocol was initiated. Thus, animals (n = 9, 10^3^ cells injected per eye and n = 3, 0 cells injected per eye) were imaged 2, 3, and 4 weeks post-injection, with weaning prior to the 4 week session. On the day of imaging, animals were transferred to the imaging suite in their home cages and sedated with dexmedetomidine (0.25–0.5 mg/kg i.p.). Immediately after sedation, 1% tropicamide was placed on the eye for dilation of the pupils, followed 1–2 minutes later by topical application of hypromellose ophthalmic demulcent solution (Gonak; Akorn, Lake Forest, IL, USA) to help prevent drying of the cornea and cataract formation. Animals were then placed on a heated platform attached to a modified microscope stage that allowed movement in the X, Y, and Z planes as well as rotation around the animal’s rostral-caudal axis. All in vivo imaging (brightfield, fluorescence, and OCT) was completed using the Micron III Image Guided OCT system for rodents (Phoenix Research Labs; Pleasanton, CA, USA). All animals had brightfield and EGFP fluorescence imaging completed and images captured using StreamPix software. OCT image gathering was performed with Micron OCT software. At the end of each imaging session, sedation was reversed by an injection of atipamezole (1 mg/kg i.p.).

### Ex vivo Analyses

Upon completion of the imaging protocols, the animals were overdosed with inhaled isoflurane, decapitated, and the eyes removed for preservation. Each eye was fixed in 4% paraformaldehyde at least overnight and then transferred to a 70% ethanol solution. After removal of anterior chamber and lens, P14 eyes were imaged for green fluorescence on a Typhoon FLA 9500 molecular imager (GE, Piscataway, NJ, USA) and tumor area manually delineated and densitized using the Analysis Tools module of ImageQuant TL software. Whole P29 eyes were paraffin embedded and 5–7 µm sections were obtained using a microtome. Sections were then deparaffinized with xylene and rehydrated in ethanol from 100% to 70%. Sections were stained using a standard Mayer’s Hematoxylin and Eosin protocol (Sigma, St. Louis, MO, USA). Briefly, tissue sections were placed in 0.1% Mayer’s Hematoxylin (filtered prior to use) for 5 minutes and rinsed in cool running tap H_2_O for 5 minutes. Sections were then stained with Eosin (0.5% in 95% ethanol) for 2.5 minutes. Slides were dipped in tap H_2_O and then dehydrated in increasing concentrations of ethanol (70–100%) followed by drying in xylenes before mounting with Permount (Fisher Scientific, Pittsburgh, PA, USA). Images were collected using a DM2000 microscope with a DFC310 FX digital CCD color camera (Leica, Buffalo Grove, IL, USA).

## Results and Discussion

### Generation of a Novel EGFP-luciferase Expressing Cell Line

**Figure 2:**
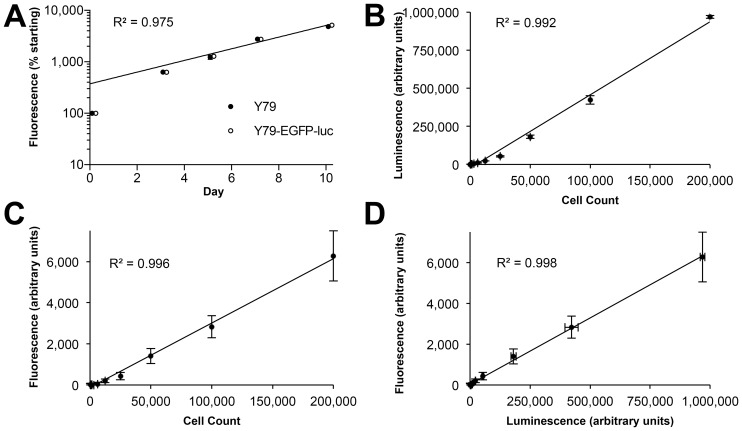
Characterization of a Y79 retinoblastoma cell line expressing an EGFP-luciferase fusion protein (Y79-EGFP-luc). (A) Y79-EGFP-luc cells display identical growth kinetics to the parent cell line (F-test p = 0.49). Y79-EGFP-luc data points shifted right for clarity. (B–D) Correlations between growth parameters: (B) Luminescence versus cell count; (C) Fluorescence versus cell count; (D) Fluorescence versus luminescence. Mean ± SD shown, n = 3 (some error bars are smaller than the data point size).

To allow both BLI and fluorescence imaging of retinoblastoma cells, we generated a derivative of the Y79 cell line, Y79-EGFP-luc, expressing a novel lentivirally-encoded EGFP-luciferase fusion protein; the modified SFFV retrovirus U3 promoter allows for high levels of EGFP-luciferase fusion protein that are maintained *in vivo*. These cells showed identical growth kinetics to the parent line (Figure 2A), indicating that insertion of the transgene did not modify the growth characteristics of the cells. Moreover, the doubling time of these cells of 64 hours (95% CI: 59–70) was similar to the value of 52 hours reported for Y79 when it was first described 40 years ago [32]. In culture, Y79-EGFP-luc cells showed excellent linearity of luciferase activity (Figure 2B) and fluorescence (Figure 2C) with increasing cell number. Importantly, these two parameters correlated closely (Figure 2D).

### Modeling of Luciferin Flux in Orthotopic Xenografts

**Figure 3:**
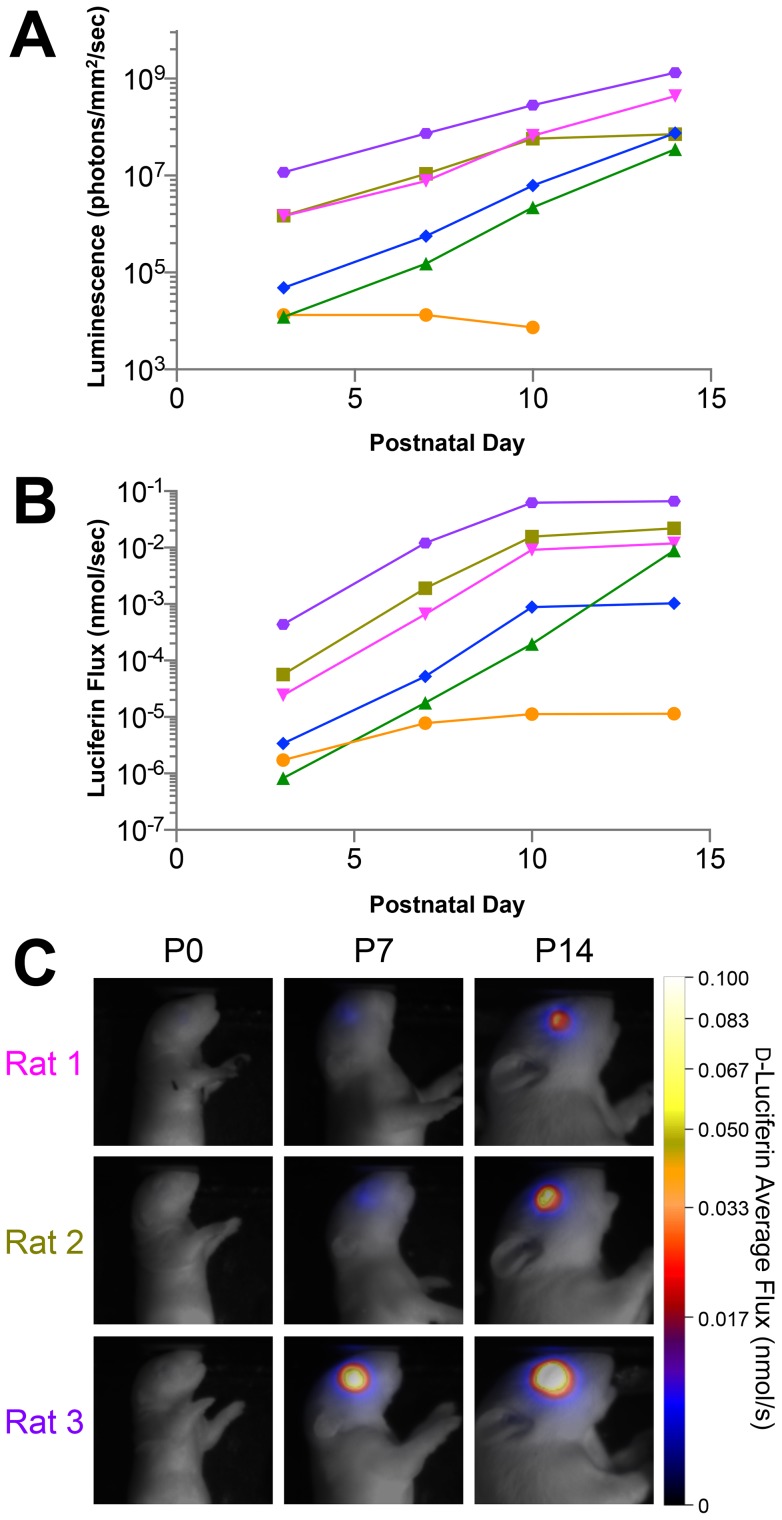
Modeling of luciferin flux from bioluminescence imaging (BLI) data reveals slowing of tumor growth over time. (A) Conventional peak luminescence imaging of xenografts in six individual animals (each animal is one colored line) shows exponential tumor growth in the majority of animals. (B) Calculated luciferin flux (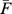) modeled (Equations 1–3) from BLI data over the 14-day study. (C) Pseudo-color parametric images of 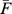 for three representative animals at three timepoints, color-coded as in (A).

We injected 10,000 Y79-EGFP-luc cells into the vitreous of P0 rats, and dynamically imaged individual animals with vitreous xenografts post administration of d-luciferin (LH_2_). Images were segmented through time using a semi-automated region of interest (ROI) tool employing a maximum entropy algorithm [34]. Peak luminescence (i.e., maximum luminescence signal over time) demonstrated exponential growth through time (Figure 3A).

We sought to determine if we could obtain more information than afforded by peak luminescence alone. To this end, we developed a new mathematical model of average luciferin flux as a more robust indicator of tumor function; this model was based on the underlying tumor bioenergetics. Key model assumptions were: 1) Metabolite production by enzymatic cleavage of the LH_2_ is observed as photon emission; 2) instantaneous LH_2_ rates follow Michaelis-Menten kinetics; and 3) pH, temperature, and quantum yield during the reaction were all constant. The quantum yield of photons from the luciferase reaction is less than perfect; for instance, up to 20% of luciferyl adenylate is involved in a non-luminescent side reaction yielding H_2_O_2_ [35]. In our model, we incorporated a previously reported quantum yield (Q_yld_) value for the luciferase reaction of 0.31 at pH 7.0 [27]. Since to our knowledge the intracellular pH of retinoblastoma cells has not been documented, we chose a constant of pH 7.0 based on an average of published values in other cell types [26], [28]. Our model also incorporated the quantum efficiency (Q_eff_) of the camera, 0.71 at 530 nm (Berthold, Oak Ridge, TN, USA) (Table 1).

**Table 1:** Parameters used for kinetic modeling of luciferin flux, 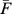.

Parameter	Value	Units	Source(s)
Q_eff_	0.71	N/A	Berthold Technologies
Q_yld_	0.31	Photons/LH_2_	[27], [47]
Eq	6.02×10^23^	LH_2_/mol	[48]
ρ	1.05	mg/mL	[49]
pH	7.0	Log[H^+^]	[26], [28]
*K_m_*	132580	nM	[50]–[53]
*V_max_*	1.63×10^14^	LH_2_/s.mg	[50]

Model estimates of tumor emission volume (*V_t_*) were computed according to the following:
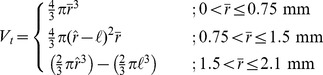
(1)Where, 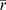 and 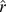 are the average radius of the ROI and the average radius of a P14 rat eye, respectively, and 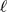 is the computed radius of a rat lens, 0.625 mm. Based on measurements of enucleated P14 eyes, we estimated the radius of the eye as 2.1 mm, and used a literature value of 1.5 mm [36] as the maximum vitreous depth, i.e., the tumor diameter above which the lens and retina would limit and mold tumor growth. Given this, 1) small tumors in the vitreous space (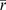
<0.75 mm) were assumed to be spherical, 2) tumors with average radii between 0.75 mm and 1.5 mm were assumed to be elliptically spherical, and 3) tumors with radii greater than 1.5 mm were assumed to be bowl-shaped following the contours of the retina and the lens. The tumor size upper bound was based on the measured radius of the P14 eye (2.1 mm).

To estimate the concentration of LH_2_ oxidized, photon luminescent emission was transformed to tumor [LH_2_] (nM) by stoichiometric and dimensional analysis using the constants in Table 1 and *V_t_* from Equation 1. The concentration of metabolite production with time can then be described by the following differential equation:
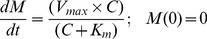
(2)Where *M* (nmol/s), *C* (nmol/L), *K_m_* (nmol/L), and *V_max_* (nmol/s) are the instantaneous rate of LH_2_ oxidized with time, the concentration of LH_2_ oxidized, the Michaelis constant for firefly luciferase, and the maximum velocity for the LH_2_/luciferase reaction, respectively. To estimate 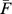 (nmol LH_2_/s), instantaneous oxidation rates were integrated with respect to the image integration period (0 to *t*, in seconds) and divided by the total integral period(s) (*T*) according to:



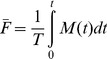
(3)A key distinction of the current model over standard approaches is that it incorporates the Michaelis-Menten kinetics of luciferase function. Luminescence directly measures the metabolic oxidation of the substrate over the image-integration period, and as such, modeling via Equations 1–3 affords a more direct physiological interpretation of the supporting biology than traditional approaches. This can be seen in Fig. 3, where peak luminescence analysis shows linear increases in tumor emission with time (Fig. 3A), while the current 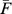 model over this same interval shows a slowing of turnover by P14 (Fig. 3B, C).

These data suggest that our new model may provide greater sensitivity to changes in cellular physiology and therefore tumor health and overall metabolism than peak luminescence alone, which simply reports information at the maximum emission. This is particularly true in the bounded environment of the eye, where tumors can reach a maximal volume and are likely to decrease metabolism once this maximum is reached.

### Ex vivo Intraocular Imaging of Xenografts

**Figure 4:**
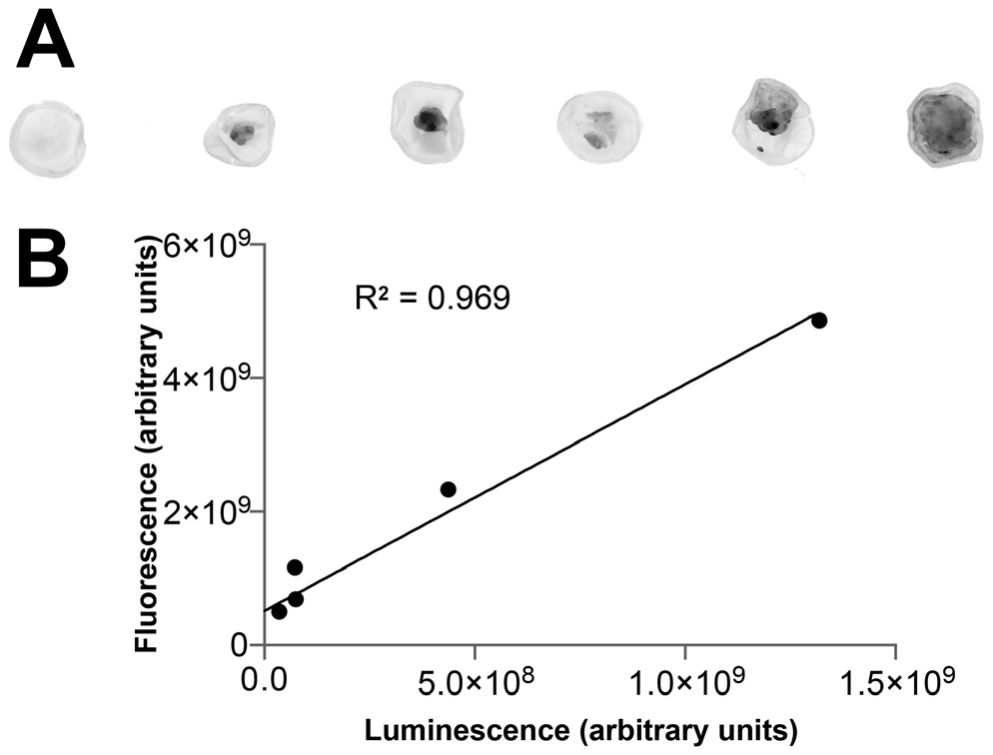
Xenograft fluorescence correlates with luminescence. (A) Ex vivo quantitative fluorescence imaging of six right eyes from six P14 rats, all of which were injected with Y79-EGFP-luc at P0. (B) Correlation between fluorescence intensity measured from individual eyes shown in (A) and peak bioluminescence.

To complement modeling of tumor function, we also analyzed the spatial development of retinoblastoma xenograft growth in our system. This important aspect of retinoblastoma xenograft biology has not been previously documented. To determine if intraocular fluorescence was correlated with tumor size, we quantified fluorescence of P14 xenografts ex vivo (Figure 4A). As with the in vitro findings (Figure 2), we confirmed that ex vivo fluorescence correlated strongly with luminescence (Figure 4B) and both increased with gross tumor area as measured ex vivo (*R* ^2^ = 0.98 and 0.95; F-test p<0.001, for fluorescence and luminescence respectively).

### In vivo Intraocular Imaging of Xenografts

**Figure 5:**
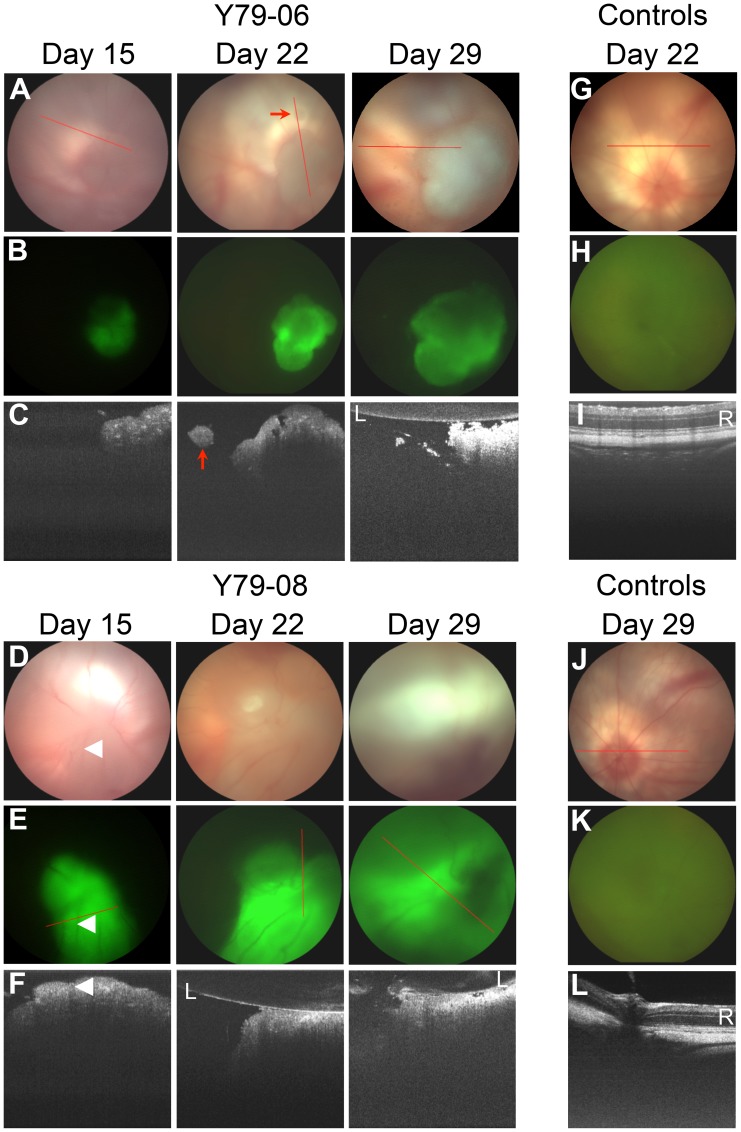
Characterization of pathology induced by Y79-EGFP-luc retinoblastoma cell xenografts or control injections. Brightfield (A,D,G,J), green fluorescence (B,E,H,K), and OCT (C,F,I,L) imaging over a 4 week period shown. Tumors were highly vascularized (*white arrowheads*) and had well-defined edges as seen on brightfield and fluorescence imaging. OCT provided some additional depth resolution not possible with the other two modalities, although this was limited by shadowing of posterior features. OCT was also able to identify small, distinct satellite tumors growing independent of the main tumor mass (*red arrows*). Red lines indicate the OCT planes; L, lens; R, retina.

In vivo, Y79-EGFP-luc xenografts were characterized by brightfield (Fig. 5A, D), green fluorescence (Fig. 5B, E), and OCT (Fig. 5C, F) over a 4 week period. All eyes (even controls) showed corneal neovascularization, likely due to the trauma of neonatal eye opening and injection; however, this pathology did not preclude imaging. Control eyes showed a normal fundus, no green fluorescence, and clear retinal OCT with no shadowing of the retina that would be suggestive of xenograft growth. (Figure 5G–L). All xenografted eyes developed tumors (9/9). Tumor size varied despite injections of equal cell numbers, which underscores the importance of BLI as a means to measure baseline tumor sizes so that tumors can be randomized accurately in future efficacy studies. On brightfield analysis, tumors were white to off-white, with prominent blood vessels, and notably dense with well-defined edges. EGFP signal was helpful in confirming the borders identified on brightfield analysis.

While both of these methods provide detailed information regarding the width of the tumor, neither provided adequate information regarding the position within the intravitreal space nor the height of the tumor. OCT allowed imaging of both normal retina adjacent to small tumors and the anterior aspect of tumors abutting the lens. However, these two planes could not be captured in a single image, so either the retina or the posterior lens was used as a visual reference point for imaging. OCT was beneficial in identifying the anterior face of the tumor and its proximity to the lens, as well as “satellite” lesions separate from the main tumor mass (Fig. 5C). In addition, large, vitreous-filling tumors that were hard to demarcate on brightfield imaging (Fig. 5D) were readily identified by OCT (Fig. 5F). OCT confirmed the presence of blood vessels within the tumor mass (Fig. 5F) as noted by brightfield (Fig. 5D) and fluorescence (Fig. 5E) imaging. Although little information on the posterior aspect of xenografts could be gleaned by OCT due to shadowing by the anterior aspect of the tumors, the ability to quickly confirm the presence and location of a xenograft in an animal eye is important. This methodology will allow the rapid profiling of multiple retinoblastoma cell lines to obtain a panel of orthotopic xenografts that can be used for therapeutic trials. Such a panel might better represent the variable genetic backgrounds and phenotypes of human tumors [2], [3].

**Figure 6:**
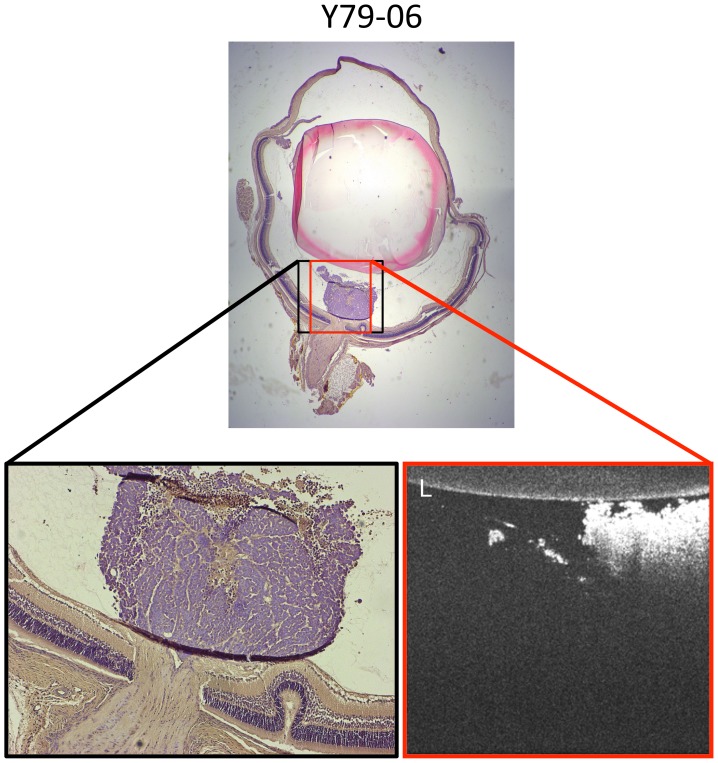
Histologic analysis of Y79 xenografts reveals intravitreal tumors with morphology closely resembling in vivo OCT imaging. On OCT imaging in(lower right), this small tumor was seen to be closely apposed to the phakic lens and was noted to have a small finger-like extension off the main tumor mass. Histologic H&E sections confirmed this same appearance after fixation. L, lens; original magnification, top = 25×, lower left = 100×.

Histologic analysis recapitulated morphology seen by OCT and confirmed preferential localization of small tumors to the intravitreal space above the optic nerve head (Fig. 6). It appeared that tumors filled the intravitreal space until coming into contact with the phakic lens, consistent with the assumptions of intravitreal tumor shape incorporated into our BLI model (Equation 1). During the one minute post-injection period, while the needle remained visualized in the vitreous space prior to withdrawal, injectate was seen to begin spreading. These cells are likely dispersed through the vitreous in the post-operative recovery period. We believe our later localization data indicate for the first time that viable Y79 retinoblastoma orthotopic xenografts likely form near the optic nerve head, where they can obtain their nutrient and blood supply from the neonatal hyaloid artery prior to its natural regression. As in other invasive tumors [37], we hypothesize that pro-angiogenic factors are released from the retinoblastoma xenografts, resulting in large, highly vascularized tumors. This assertion is supported by evidence that retinoblastoma subcutaneous xenografts depend on VEGF signaling for growth [38].

## Conclusions

There is a long history of xenografting retinoblastoma cell lines into mice, rats, and rabbits [39]; even recently zebrafish [40]. Cells and tumor material have been delivered subcutaneously [41], intravenously [13], into the anterior chamber [42], and by intravitreal, subretinal, and subconjunctival routes [13]. Traditionally, successful orthotopic xenografts were confirmed by fundus photography, signs such as progressive proptosis seen on examination as tumors grow, and postmortem histopathology. Recently, prior to xenografting into rodents, retinoblastoma cell lines have been genetically manipulated to express EGFP, luciferase, or a dual construct of both (EGFP-luc) as methods to follow tumor growth [12]–[14], [16], [17], [43]. More rapid and quantitative ascertainment of xenograft success and growth would be advantageous.

For the first time, we have shown that a Y79-EGFP-luc cell line creates viable xenografts in the newborn rat model. Further, we have shown that the ability to monitor xenograft growth spatially in the eye as well as model tumor function offers considerable utility for future therapeutic trials. Subtle readouts of compound activity, such as diffusion, thinning, or movement of the tumor mass, loss of xenograft core fluorescence, and decreased 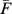 are likely to provide sensitive measures of the efficacy of experimental agents, even when gross effects on tumor volume or peak luminescence are modest. In addition, our imaging methodologies complement what is possible in transgenic retinoblastoma models, one of which has previously been examined using OCT [44]–[46]. Transgenic models have the advantage of intraretinal tumors more readily documented by OCT without shadowing, but lack the human cell origin and ability to quantitatively follow tumor growth by BLI as in the current system.

Therefore, we believe the current model greatly enhances the traditional approaches, and will provide additional key information necessary for future studies of novel therapeutics in retinoblastoma. Moreover, this system allows us to rapidly expand preclinical xenograft studies to additional retinoblastoma cell lines and move beyond the aggressive Y79 cell line commonly used.
